# A Cross-Sectional Study on the Observation of Clinical Profiles and Associated Electrolyte Disturbances in Patients Admitted to the Pediatric Intensive Care Unit (PICU) at a Tertiary Care Center

**DOI:** 10.7759/cureus.77698

**Published:** 2025-01-20

**Authors:** Upendra Prasad Sahu, Md Rizwan Farooquee, Omar Hasan, Sameen Ehtesham, Riaz Hasan

**Affiliations:** 1 Department of Pediatrics, Rajendra Institute of Medical Sciences, Ranchi, IND; 2 Department of Chemistry, Dr Shyama Prasad Mukherjee University, Ranchi, IND

**Keywords:** cns disorders, critically ill children, electrolyte disturbances, hypocalcemia, hypokalemia, hypomagnesemia, hyponatremia, pediatric intensive care unit, respiratory disorders

## Abstract

Objective: This study aims to determine the prevalence of electrolyte disturbances in children hospitalized in the pediatric intensive care unit (PICU) of Rajendra Institute of Medical Sciences (RIMS), a tertiary care center in Ranchi, India, and to evaluate related factors like pneumonia, heart disease, meningitis/encephalitis, and others.

Methods: Serum electrolyte levels (sodium, potassium, calcium, magnesium) were obtained from 110 patients admitted to the PICU at RIMS. Statistical analyses were conducted to identify the prevalence and association of disturbances with specific diseases.

Results: Sodium disturbances was most common (32 cases, 29%), with 28 (25.45%) patients exhibiting hyponatremia and four exhibiting hypernatremia (3.63%). Hypokalemia (10 cases, 9.09%) was associated predominantly with central nervous system (CNS) disorders. Hypocalcemia (26 cases, 23.63%) was most frequently linked to respiratory disorders. Hypomagnesemia cases (18, 16.36%) were also prevalent.

Conclusion: Electrolyte disturbances are common in critically ill pediatric patients and are associated with various systemic disorders, emphasizing the need for regular monitoring in the PICU.

## Introduction

Electrolyte disturbances are common in hospitalized children in the pediatric intensive care unit (PICU) due to underlying diseases and specific medications. Common electrolyte imbalances include sodium, potassium, calcium, and magnesium disorders [1,2]. Sodium disturbances, such as hypernatremia (20%-30% prevalence in PICU) [3-7], is often iatrogenic and associated with factors like sodium bicarbonate use, diuretics, and fluid losses. Hypernatremia may also occur in diabetes insipidus and with drugs like lithium and amphotericin B [3-7]. Hyponatremia is linked to abnormal vasopressin production, diuretics, and kidney damage, with severe cases leading to cerebral edema and complications like seizures or coma [8,9].

Potassium disturbances include hypokalemia caused by decreased intake, sympathomimetics, insulin, diuretics, or amphotericin B, leading to symptoms like muscle weakness, paralysis, and cardiac arrhythmias [10]. Hyperkalemia arises from renal and adrenal insufficiency, insulin resistance, tissue damage, or medications like beta blockers and nonsteroidal anti-inflammatory drugs (NSAIDs). Severe hyperkalemia can result in fatal arrhythmias [11]. Hypocalcemia is seen in up to 90% of PICU children, with ionized hypocalcemia affecting 15%-20%. Causes include trauma, renal failure, sepsis, vitamin D deficiency, and hypoparathyroidism. Symptoms include muscle spasms, neurological issues, and cardiac effects such as prolonged QT intervals [12]. Hypomagnesemia, though often asymptomatic, is linked to gastrointestinal and renal losses and drugs like amphotericin and cisplatin. Severe cases present with symptoms of hypocalcemia, including muscle spasms and seizures, with levels below 1.5 mg/dL indicating hypomagnesemia [13].

Despite the well-documented global prevalence of electrolyte disturbances, there is a paucity of region-specific data from resource-limited settings, particularly in our region. The distinct demographic, dietary, and healthcare factors in our area may influence the incidence and etiology of these imbalances. This knowledge gap prompted our research to enhance our understanding of the clinical profiles and related electrolyte disturbances in pediatric patients admitted to our tertiary care PICU. We aim to identify prevalent patterns and their correlations with systemic disorders to enhance management strategies tailored to the specific needs of critically ill children in this context.

## Materials and methods

This hospital-based cross-sectional observational study was conducted to evaluate the prevalence and associated etiology of electrolyte disturbances in critically ill pediatric patients. The study took place in the PICU at the Department of Pediatrics from June 2021 to May 2022 at Rajendra Institute of Medical Sciences (RIMS) in Ranchi, Jharkhand, India, a tertiary care center catering to a diverse population. A total of 110 pediatric patients admitted to the PICU during the study period were included. Convenience sampling was employed to enroll all eligible patients who fulfilled the inclusion criteria, which included pediatric patients admitted to the PICU with informed consent obtained from their parents or guardians. Patients with a history of chronic renal disorders, chronic diarrhea, or vomiting prior to hospitalization, or pre-existing electrolyte disturbances documented before PICU admission, were excluded from the study. Limitations included the inability to obtain serum magnesium levels for all patients due to logistical constraints, and such cases were excluded from the analysis [14-15].

Detailed clinical histories were documented for all enrolled patients, followed by comprehensive physical examinations and any routine and necessary laboratory investigations. Venous blood samples were collected within 24 hours of admission to measure serum levels of sodium, potassium, calcium, and magnesium. Laboratory analyses included ion-selective electrode methods for sodium, potassium, and calcium, and Cobas Integra automated analyzers for magnesium [16-18]. The reference ranges for electrolyte disturbances are presented (Table 1).

**Table 1:** Reference ranges for electrolyte disturbances

Electrolyte disturbance	Reference range
Hyponatremia	Sodium < 135 mEq/L
Hypernatremia	Sodium > 145 mEq/L
Hypokalemia	Potassium < 3.5 mEq/L
Hyperkalemia	Potassium > 5.5 mEq/L
Hypocalcemia	Calcium < 9 mg/dL
Hypercalcemia	Calcium > 10.5 mg/dL
Hypomagnesemia	Magnesium < 1.5 mEq/L
Hypermagnesemia	Magnesium > 2.6 mEq/L

Data were compiled and analyzed using Microsoft Excel (Microsoft Corp., Redmond, WA) and IBM SPSS Statistics software version 21.0 (IBM Corp., Armonk, NY). Descriptive statistics, such as frequencies and percentages, were used to summarize categorical data. The chi-square test was employed to evaluate associations between electrolyte disturbances and clinical outcomes, with a p-value of < 0.05 considered statistically significant. To ensure data accuracy and reliability, laboratory tests were performed by trained personnel following standard operating procedures, with regular equipment calibration. Data entry was double-checked to minimize errors.

The study was approved by the Institutional Ethics Committee of RIMS (approval number: MEMO NO-311, dated 05/07/2021). Written informed consent was obtained from the parents or guardians of all participants.

## Results

Sodium disturbances

**Figure 1:**
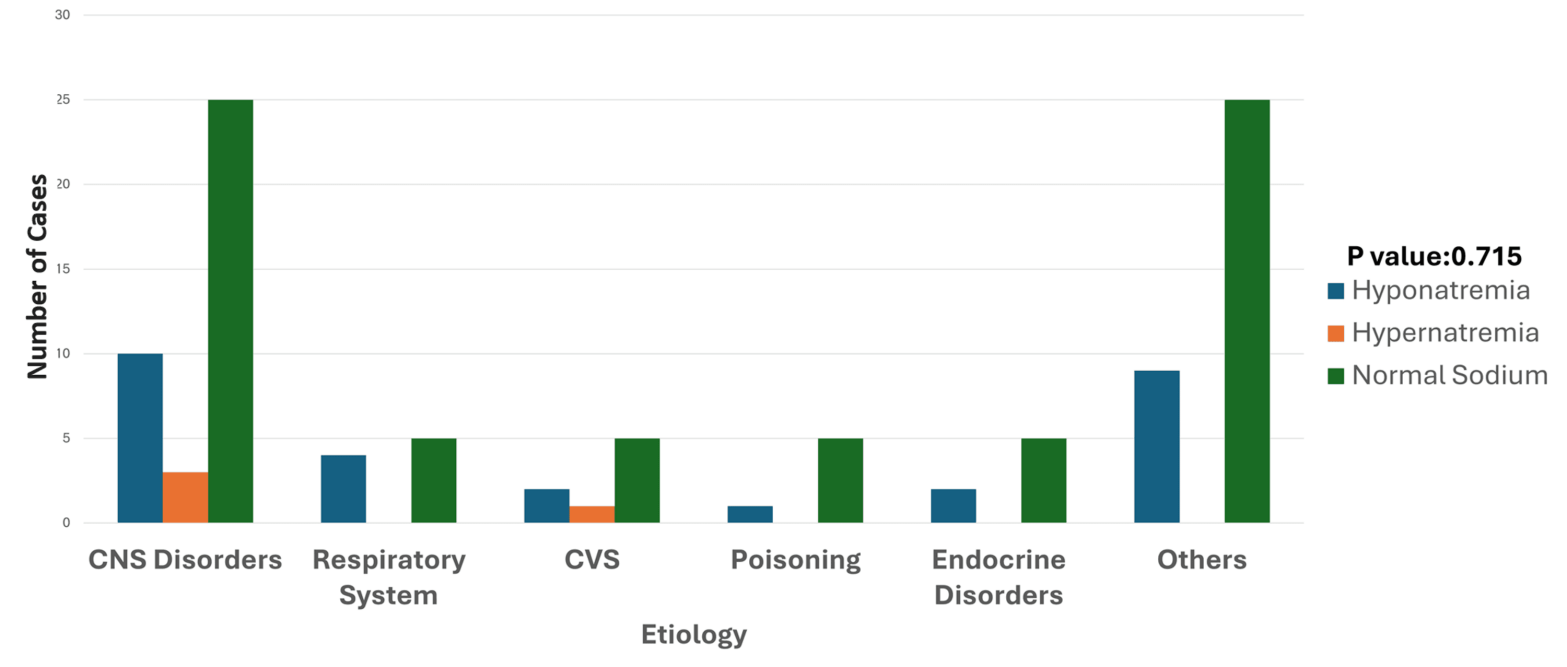
Etiology and sodium disturbances Hyponatremia is most prevalent in CNS disorders, followed by the "Others" category, and is relatively low in respiratory, CVS, and poisoning cases. Hypernatremia is rare, with a small presence observed primarily in CNS disorders. Normal sodium levels dominate across all categories, particularly in respiratory and "Others" groups. The p-value was calculated between etiologies and the sodium disturbance categories (hyponatremia, hypernatremia, and normal sodium levels). Significant p-value: <0.05 CNS: central nervous system; CVS: cardiovascular system

**Figure 2:**
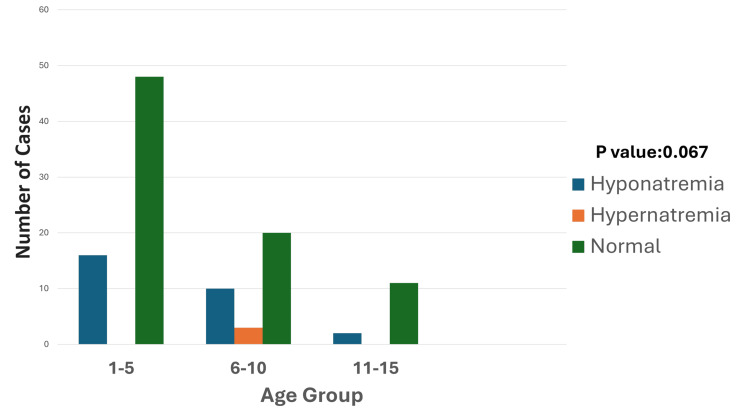
Age-wise distribution of sodium disturbances The graph illustrates sodium levels across age groups, showing that hyponatremia is most prevalent in the one- to five-year age group (16 cases), followed by the six- to 10-year age group (10 cases), and is least common in the 11- to 15-year age group (two cases). Hypernatremia is rare, with only three cases reported in the six- to 10-year-old age group. Most patients across all age groups had normal sodium levels, particularly in the one- to five-year group (48 patients). These findings highlight that younger children are more vulnerable to sodium disturbances, especially hyponatremia. The p-value was calculated by comparing the prevalence of hyponatremia, hypernatremia, and normal sodium levels across age groups (one to five years, six to 10 years, and 11 to 15 years). Significant p-value: <0.05

Sodium disturbances, encompassing both hyponatremia and hypernatremia, were observed among the study population, highlighting their potential clinical implications in critically ill pediatric patients. Hyponatremia was identified in 28 patients (25.4%), with the highest prevalence noted in those with central nervous system (CNS) disorders such as tubercular meningitis, hydrocephalus, and encephalitis, in 10 cases (35.7%), followed by respiratory disorders including pneumonia and bronchiolitis in four cases (14.2%) (Figure 1). A detailed age-based analysis revealed that hyponatremia predominantly affected the one- to five-year age group (16 cases), with fewer cases in the six- to 10-year (10 cases) and 11- to 15-year (two cases) age groups (Figure 2).

Hypernatremia, on the other hand, was less common, occurring in four patients (3.6%), with CNS disorders such as cerebral edema and seizures secondary to infections accounting for three (75%) of the cases. Most hypernatremia cases were seen in the six- to 10-year age group, with three cases (Figure 2), and a single case was reported in the one- to five-year age group.

Despite the clinical importance of sodium disturbances in critically ill children, statistical analysis revealed no significant association between these disturbances and mortality (p = 0.24). This finding underscores the multifactorial nature of mortality in PICU settings, where electrolyte disturbances are just one of many contributing factors. The prevalence of sodium disturbances in CNS disorders, in particular, may reflect the pathophysiological interplay between the neurological system and sodium homeostasis, emphasizing the need for vigilant monitoring and management in this subgroup.

Potassium disturbances

**Figure 3:**
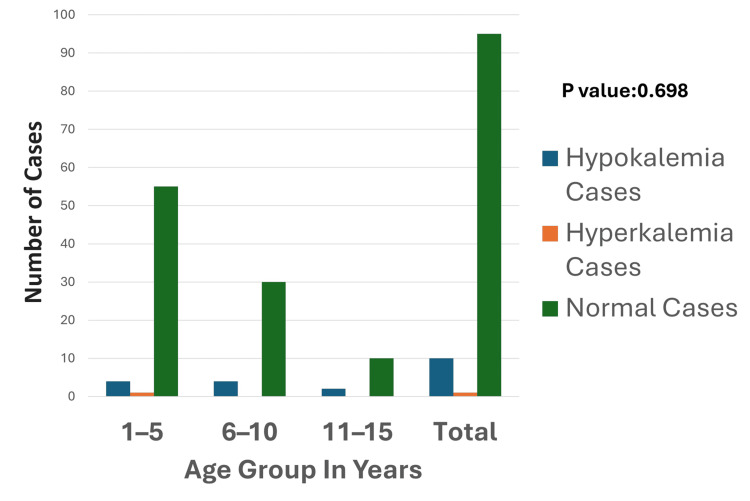
Age-wise distribution of potassium disturbances The graph highlights the distribution of potassium levels across different age groups, demonstrating that hypokalemia is most prevalent in the one- to five-year age group, with a smaller number of cases observed in older age groups. Hyperkalemia is rare, with minimal representation only in the one- to five-year age group. Normal potassium levels dominate across all age groups, particularly in the one- to five-year age group. The p-value was calculated by comparing the prevalence of hypokalemia, hyperkalemia, and normal potassium levels across age groups (one to five years, six to 10 years, and 11 to 15 years). Significant p-value: <0.05

**Figure 4:**
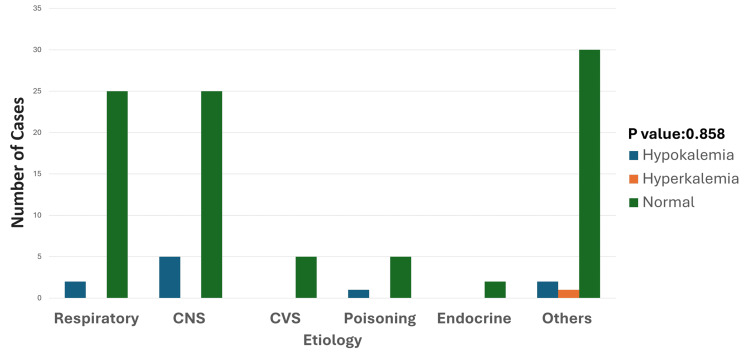
Etiology and potassium disturbances Among the 10 patients with hypokalemia, the most common etiology was CNS-related conditions (seizures, CNS infections), accounting for five cases (50%). Other causes included respiratory disorders (two cases, 20%), poisoning (one case, 10%), and "others" (two cases, 20%). Hyperkalemia was rare, with only one case observed in the "others" category, associated with hepatic encephalopathy. The p-value was calculated by comparing the prevalence of hypokalemia and hyperkalemia across different etiologies, including CNS-related conditions, respiratory disorders, poisoning, and miscellaneous causes. Significant p-value: <0.05 CNS: central nervous system; CVS: cardiovascular system

Out of 110 children studied, 11 cases (10%) were found to have potassium abnormalities, comprising 10 cases (9.09%) of hypokalemia and one case (0.9%) of hyperkalemia. Among the 10 cases of hypokalemia, four cases each were observed in the age groups of one to five years and six to 10 years, while two cases were from the age group of 11 to 15 years (Figure 3). A single case of hyperkalemia was observed in the one-to-five-year age group. Regarding the etiology of hypokalemia, the most common cause was CNS-related conditions, such as seizures and CNS infections, accounting for five cases (50%). Other causes included respiratory conditions (two cases, 20%); poisoning (one case, 10%); and miscellaneous causes (two cases, 20%) (Figure 4). The sole case of hyperkalemia was categorized under miscellaneous causes and was associated with hepatic encephalopathy.

The clinical implications of potassium disturbances are considerable, as they can lead to life-threatening complications such as cardiac arrhythmias. Hypokalemia, in particular, has been associated with neuromuscular weakness and an increased risk of mortality in critically ill patients, underscoring the critical need for careful monitoring and timely intervention. However, statistical analysis in this study did not demonstrate a significant association between potassium levels and mortality (p > 0.05), suggesting that while important, potassium disturbances may be part of a broader spectrum of factors influencing outcomes in the PICU. These findings emphasize the necessity for comprehensive management strategies that address both electrolyte imbalances and their underlying causes.

Calcium disturbances

**Figure 5:**
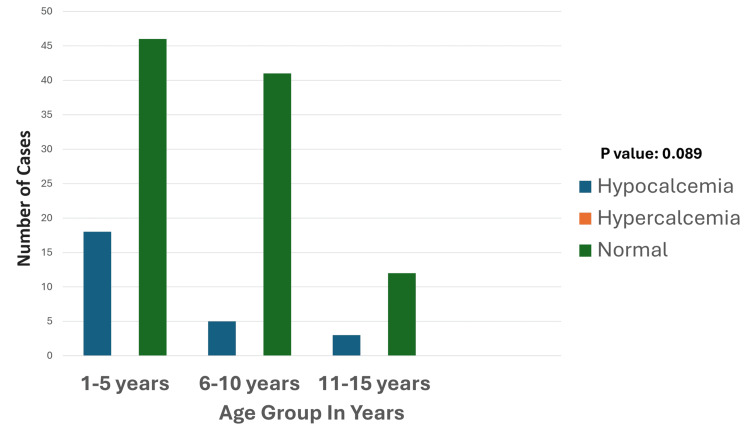
Age-wise distribution of calcium disturbances The graph highlights the distribution of calcium levels across different age groups, showing that hypocalcemia is most prevalent in the one- to five-year age group, with fewer cases observed in the six- to 10-year age group and 11- to 15-year age groups. Hypercalcemia is notably absent across all age categories. Normal calcium levels are predominant, particularly in the 1one- to five-year age group, followed by the six- to 10-year age group, and 11- to 15-year age groups. The p-value was calculated by comparing the prevalence of hypocalcemia and normal calcium levels across age groups (one to five years, six to 10 years, and 11 to 15 years). Significant p-value: <0.05

Among the 110 pediatric patients studied, hypocalcemia emerged as a notable electrolyte disturbance, affecting 26 cases (23.6%). Hypercalcemia, however, was not observed in any of the cases. Hypocalcemia was most frequently associated with respiratory disorders, accounting for 11 cases (42.3%). Age distribution analysis revealed that the one- to five-year age group was disproportionately affected, comprising 18 of the 26 cases (Figure 5). Additionally, hypocalcemia showed a higher prevalence in males (11 cases, 65.3%) compared to females (seven cases, 34.6%).

**Figure 6:**
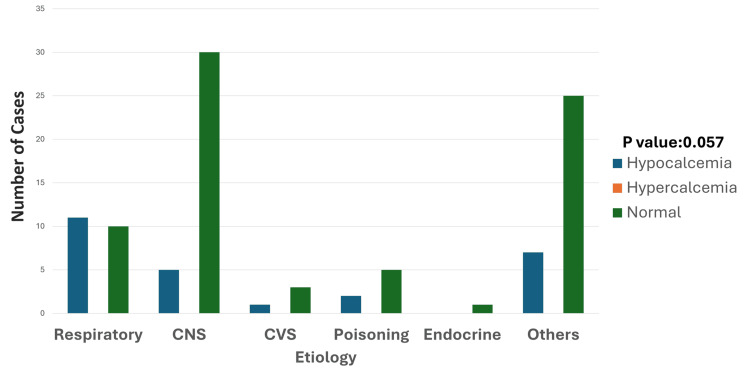
Etiology and calcium disturbances Hypocalcemia is most frequently observed in respiratory and "others" categories, with a smaller presence in CNS, CVS, and poisoning conditions. Hypercalcemia is absent across all categories. Normal calcium levels predominate in all conditions, particularly in the CNS and "others" groups. The p-value was calculated by comparing the prevalence of hypocalcemia across different etiologies, including respiratory disorders, CNS conditions, CVS conditions, poisoning, and miscellaneous causes. Significant p-value: <0.05 CNS: central nervous system; CVS: cardiovascular system

Among 26 patients with hypocalcemia, the most number of patients were from the respiratory system; 11 cases (42.3%) accounted for all hypocalcemia cases, CNS, cardiovascular system (CVS), poisoning, and others (snake bite, viral hemorrhagic fever, malaria) had five (19.2%), one (3.8%), two (7.6%), and seven cases (26.9%) of hypocalcemia, respectively (Figure 6).

Neurological symptoms such as muscle cramps and seizures were commonly reported in patients with hypocalcemia, reflecting the clinical impact of calcium disturbances on neuromuscular function. Statistical analysis suggested a potential association between hypocalcemia and prolonged stays in the PICU, although further research is needed to substantiate this link. These findings highlight the importance of vigilant calcium monitoring and management, particularly in young children with respiratory disorders or those presenting with neurological symptoms, to mitigate complications and improve clinical outcomes.

Magnesium disturbances

**Figure 7:**
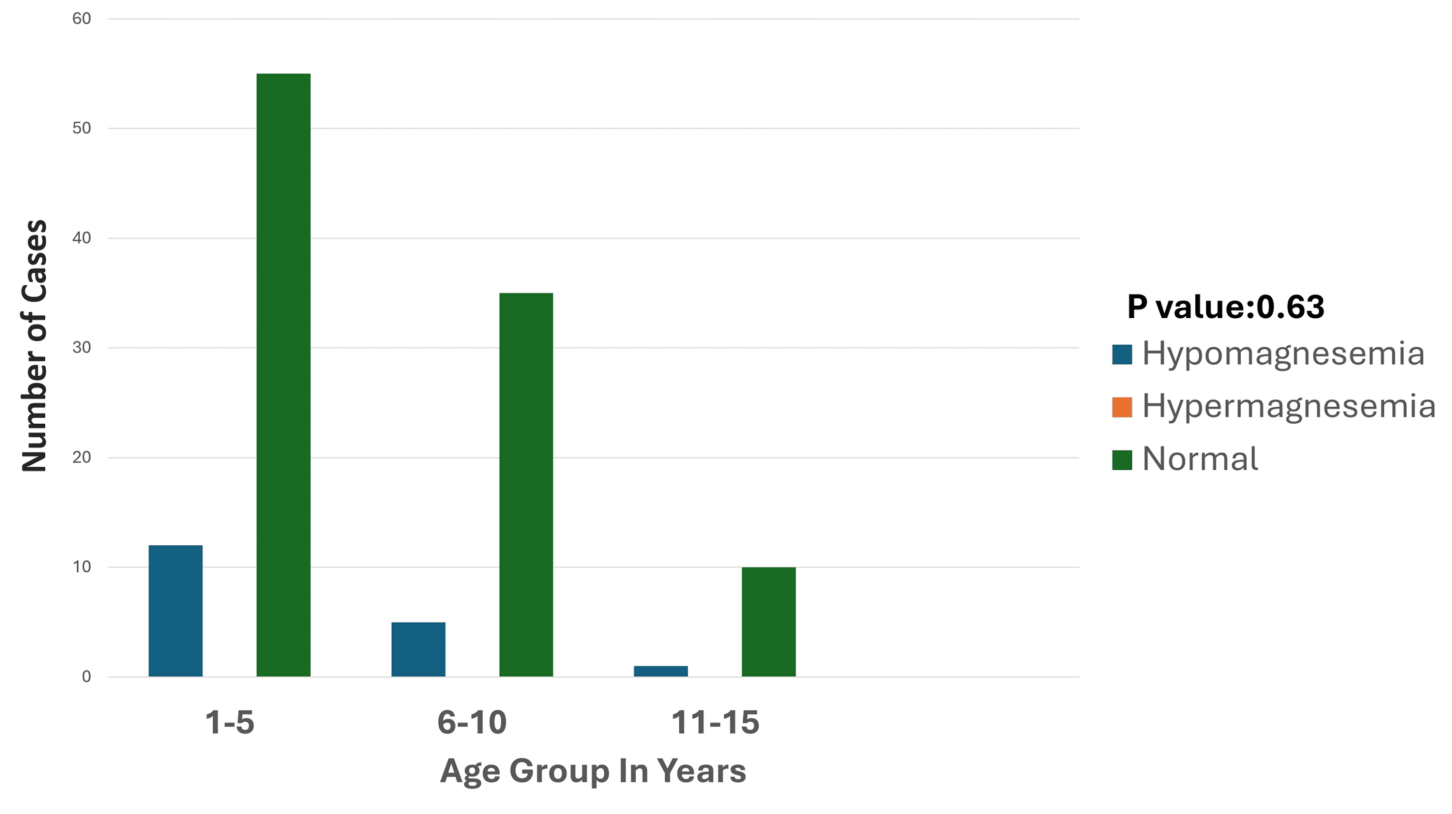
Age-wise distribution of magnesium disturbances Hypomagnesemia is most prevalent in the one- to five-year age group, with fewer cases in the six- to 10- and 11- to 15-year age groups. Hypermagnesemia is not observed in any age group. Normal magnesium levels dominate across all groups, particularly in the one- to five- and six- to 10-year age groups. The p-value was calculated by comparing the prevalence of hypomagnesemia and normal magnesium levels across age groups (one to five years, six to 10 years, and 11 to 15 years). Significant p-value: <0.05

Hypomagnesemia was identified in 18 cases (16.3%), with respiratory disorders emerging as the leading associated condition, accounting for five cases (27.7%). The prevalence of hypomagnesemia was notably higher in the one- to five-year age group, comprising 12 cases (66.6%) of the affected patients (Figure 7), suggesting a particular vulnerability among younger children.

**Figure 8:**
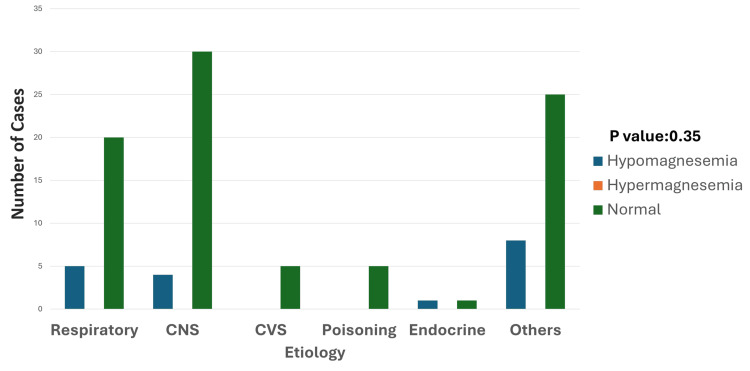
Etiology and magnesium disturbances Hypomagnesemia is most frequently observed in the "others" category, followed by respiratory and CNS conditions, with fewer cases seen in endocrine disorders. Hypermagnesemia is absent across all categories. Normal magnesium levels are predominant in all conditions, especially in the CNS and "others" groups. The p-value was calculated by comparing the prevalence of hypomagnesemia across different etiologies, including respiratory disorders, CNS conditions, endocrine disorders, and miscellaneous causes. Significant p-value: <0.05 CNS: central nervous system; CVS: cardiovascular system

Out of 18 cases of hypomagnesemia, five (27.7%) were from the respiratory system. Four (22.2%) were from the CNS. One case (5.5%) was from the endocrine system, and eight (44.4%) were from the "others" category (Figure 8).

Clinically, magnesium disturbances presented with symptoms such as muscle weakness and spasms, which often complicated the course of the illness and required additional management strategies. Although no statistically significant association between hypomagnesemia and mortality was observed, its presence was linked to prolonged stays in the PICU, highlighting its impact on recovery and resource utilization. 

## Discussion

**Figure 9:**
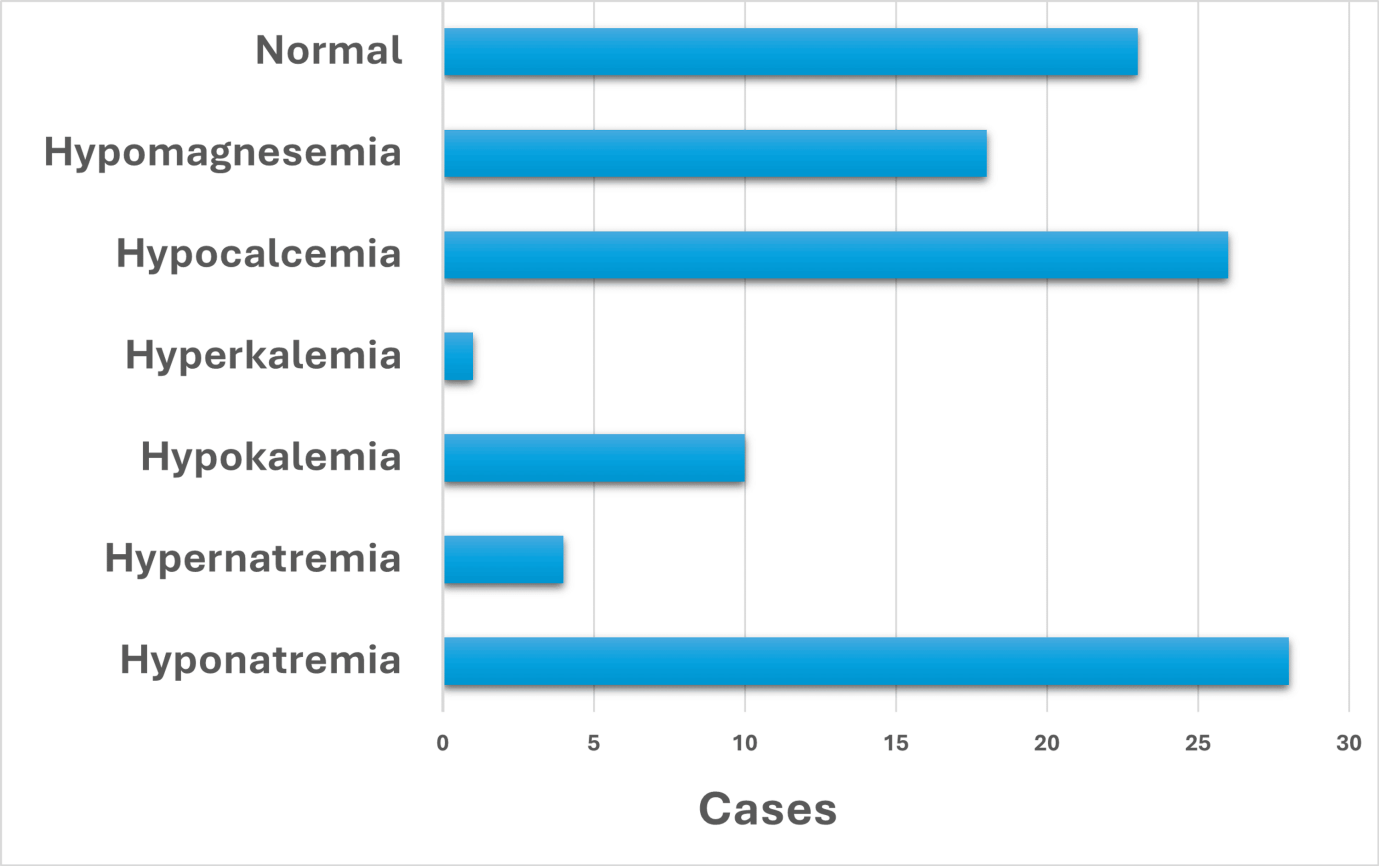
Distribution of electrolyte disturbances The bar graph illustrates the distribution of electrolyte levels among patients, showing that the majority had normal values. Among the electrolyte imbalances, hyponatremia was the most common, followed by hypocalcemia and hypomagnesemia. Hypokalemia was observed in a moderate number of cases, while hyperkalemia and hypernatremia were rare, with very few occurrences.

This study underscores the high prevalence of electrolyte disturbances (Figure 9) among critically ill pediatric patients in the first 24 hours of PICU admission in our cohort. 

Electrolyte disturbances are among the most common clinical challenges encountered in PICUs, often associated with significant systemic complications. This study highlights the prevalence and impact of sodium, potassium, calcium, and magnesium disturbances in critically ill children, emphasizing their clinical implications and the need for routine monitoring.

Sodium disturbances

Sodium disturbances were the most prevalent, and predominantly associated with CNS disorders. Hyponatremia, observed in 28 cases (25.4%), was most common in patients with conditions such as tubercular meningitis, encephalitis, and hydrocephalus. These conditions are known to cause hyponatremia through mechanisms such as the syndrome of inappropriate antidiuretic hormone secretion (SIADH) [2,19]. Younger children (one to five years) were particularly affected, reflecting their vulnerability to fluid and electrolyte imbalances due to their limited physiological reserves. Hyponatremic patients presented with symptoms ranging from lethargy and altered sensorium to seizures, underscoring the risks of cerebral edema and neurological complications. Although no direct association with mortality was found, the clinical severity of hyponatremia warrants vigilant monitoring and prompt management to prevent adverse outcomes. Hypernatremia, identified in four cases (3.6%), was primarily linked to CNS disorders such as brain injury and dehydration secondary to inadequate fluid intake. The clinical presentation included irritability, hyperreflexia, and altered mental status, emphasizing the importance of careful fluid management.

Potassium disturbances

Potassium disturbances, though less common, were clinically significant. Hypokalemia was observed in 10 cases (9.1%), often associated with CNS disorders, including seizures and meningitis, as well as respiratory conditions like pneumonia. The symptoms of hypokalemia included muscle weakness, hypotonia, and, in severe cases, arrhythmias. These findings align with previous studies highlighting the importance of potassium homeostasis in reducing mortality and morbidity in critically ill children [20]. Risk factors such as diuretic use, corticosteroid therapy, and gastrointestinal losses were notable contributors, necessitating proactive monitoring and timely intervention [21]. Hyperkalemia, though rare in this cohort, poses a significant risk of fatal arrhythmias and should be closely monitored in patients with renal dysfunction or tissue damage.

Calcium disturbances

Hypocalcemia was notably prevalent, affecting 26 cases (23.6%), with respiratory disorders such as pneumonia and acute respiratory distress syndrome (ARDS) being the most common etiologies. Hypocalcemia frequently presents with neuromuscular symptoms such as tetany, laryngospasm, and seizures, as well as cardiovascular manifestations, including prolonged QT intervals and hypotension. These findings are consistent with literature reporting hypocalcemia as a significant contributor to cardiac dysfunction and prolonged PICU stays [19]. The higher prevalence in males and its association with metabolic stress and inflammation highlight areas for further investigation. Ionized calcium measurement is recommended for more accurate clinical correlations, as it better reflects the physiologically active calcium fraction [22].

Magnesium disturbances

Magnesium disturbances, primarily hypomagnesemia, were observed in 18 cases (16.3%), with respiratory and CNS disorders being the leading causes. The younger age group (one to five years) was particularly susceptible, presenting with neuromuscular symptoms such as tremors, seizures, and weakness. Although no direct link to mortality was observed, hypomagnesemia correlated with prolonged hospital stays and increased susceptibility to sepsis and poor wound healing, as supported by existing studies [19,7]. This underscores the importance of routine magnesium monitoring and early correction to prevent complications.

Prolonged PICU stays and the role of emerging technologies

Prolonged PICU admissions are often influenced by factors such as electrolyte imbalances, which complicate management and delay recovery. The frequent occurrence of disturbances like hyponatremia, hypokalemia, and hypocalcemia in critically ill children necessitates their early identification and treatment [14,15]. Emerging technologies, including machine learning (ML) and artificial intelligence (AI), present transformative opportunities in this domain; ML models have shown promise in improving mortality prediction and real-time electrolyte monitoring in critically ill pediatric patients [23]. These tools can analyze large datasets to identify subtle trends, predict electrolyte imbalances, and provide timely alerts to clinicians, enabling proactive interventions [24]. Integrating these technologies into PICU workflows could optimize resource utilization, alleviate the burden of prolonged stays, and enhance patient outcomes [25].

Study limitations

This study provides valuable insights into the prevalence and implications of electrolyte disturbances in PICU settings; however, several limitations must be acknowledged. First, the single-center design limits the generalizability of findings to other settings with different patient populations and resource availability. Second, the cross-sectional design precludes the assessment of temporal changes or causal relationships. Third, incomplete magnesium measurements due to logistical constraints may have introduced selection bias. Finally, the study did not account for potential confounding factors, such as concurrent medications, nutritional status, or comorbidities, which may have influenced electrolyte disturbances.

## Conclusions

This study highlights the significant prevalence of electrolyte disturbances among critically ill pediatric patients on the first day of PICU admission, with sodium disturbances being the most common, followed by calcium, magnesium, and potassium imbalances. The association of these disturbances with underlying conditions such as CNS and respiratory disorders emphasizes the need for regular monitoring and timely interventions. Although mortality rates were not directly correlated with most disturbances, their role in prolonging hospital stays and complicating recovery underscores their clinical importance. Future research with a larger, multicenter cohort and a longitudinal design could provide deeper insights into causative factors and long-term outcomes, enabling more effective management strategies for these critical conditions. Electrolyte disturbances are common in critically ill pediatric patients and are associated with various systemic disorders, emphasizing the need for regular monitoring in the PICU.
